# State Strategies to Address Opioid Use Disorder Among Pregnant and Postpartum Women and Infants Prenatally Exposed to Substances, Including Infants with Neonatal Abstinence Syndrome

**DOI:** 10.15585/mmwr.mm6836a1

**Published:** 2019-09-13

**Authors:** Charlan D. Kroelinger, Marion E. Rice, Shanna Cox, Hadley R. Hickner, Mary Kate Weber, Lisa Romero, Jean Y. Ko, Donna Addison, Trish Mueller, Carrie Shapiro-Mendoza, S. Nicole Fehrenbach, Margaret A. Honein, Wanda D. Barfield

**Affiliations:** ^1^Division of Reproductive Health, National Center for Chronic Disease Prevention and Health Promotion, CDC; ^2^CDC Foundation, Atlanta, Georgia; ^3^Division of Congenital and Developmental Disorders, National Center on Birth Defects and Developmental Disabilities, CDC.

Since 1999, the rate of opioid use disorder (OUD) has more than quadrupled, from 1.5 per 1,000 delivery hospitalizations to 6.5 (*1*), with similar increases in incidence of neonatal abstinence syndrome (NAS) observed for infants (from 2.8 per 1,000 live births to 14.4) among Medicaid-insured deliveries (*2*). CDC’s response to the opioid crisis involves strategies to prevent opioid overdoses and related harms by building state capacity and supporting providers, health systems, and payers.* Recognizing systems gaps in provision of perinatal care and services, CDC partnered with the Association of State and Territorial Health Officials (ASTHO) to launch the Opioid Use Disorder, Maternal Outcomes, and Neonatal Abstinence Syndrome Initiative Learning Community (OMNI LC). OMNI LC supports systems change and capacity building in 12 states.^†^ Qualitative data from participating states were analyzed to identify strategies, barriers, and facilitators for capacity building in state-defined focus areas. Most states focused on strategies to expand access to and coordination of quality services (10 of 12) or increase provider awareness and training (nine of 12). Fewer states focused on data, monitoring, and evaluation (four of 12); financing and coverage (three of 12); or ethical, legal, and social considerations (two of 12). By building capacity to strengthen health systems, state-identified strategies across all focus areas might improve the health trajectory of mothers, infants, and families affected by the U.S. opioid crisis.

Guidance for pregnant and postpartum women with OUD includes universal screening for substance use during pregnancy; provision of medication-assisted treatment and behavioral counseling during pregnancy and the postpartum period; anticipation and management of NAS for infants prenatally exposed to substances; and multidisciplinary, long-term follow-up care for mothers and infants to improve outcomes.^§^ Provision of services requires coordinated effort among providers, health departments, and other state and local agencies, including residential treatment programs, housing authorities, and child welfare agencies.^¶^ OMNI LC uses a learning collaborative framework (*3*) that is designed to support states in developing and implementing systems change on complex public health issues.

As part of the learning collaborative framework, 12 state teams, comprising leaders from multidisciplinary agencies,** participated in a 2-day meeting in Arlington, Virginia, in November 2018, with support from ASTHO, CDC, and other federal and academic partners.^††^ Five focus areas were defined: 1) access to and coordination of quality services; 2) provider awareness and training; 3) data, monitoring, and evaluation; 4) financing and coverage; and 5) ethical, legal, and social considerations. State teams developed plans of action within one or more focus areas and outlined activities to accomplish goals. CDC abstracted data from state action plans and other information sources (i.e., topic-specific discussion notes and state presentations). CDC coded data and identified strategies, existing barriers, and facilitators.^§§^ Codes were validated by a separate group of CDC researchers using the same codebook; differences were resolved through consensus.

## Focus Areas

**Access to and coordination of quality services**. Among the 12 state teams, 10 developed action plans to address access to and coordination of quality services for pregnant and postpartum women with OUD and infants prenatally exposed to substances, including infants with NAS (Table 1). Existing barriers included geographic and logistic challenges (e.g., limited resources in rural areas and lack of transportation or child care) and lack of coordinated clinical and social services (Table 2). Strategies included coordination of OUD treatment, wraparound services (e.g., nutrition or mental health services), and trauma-informed, family-centered care; improvement in collaboration between state agencies and other state organizations; and implementation of statewide perinatal quality collaboratives (Table 3). Telemedicine could facilitate access to care in rural areas or areas with limited services (Table 2).

**TABLE 1:** Defined areas of focus targeting pregnant and postpartum women with opioid use disorder and infants prenatally exposed to substances, by state — Opioid Use Disorder, Maternal Outcomes, and Neonatal Abstinence Syndrome Initiative Learning Community, 2018

Focus area	Definition	State											
		AK	FL	IL	KY	NV	OH	PA	RI	TN	VT	WA	WV
Access to and coordination of quality services	Assessment of eligibility and availability of services to aid in treatment, referral, or recovery efforts (e.g., mental health services, child care, and transportation services), coordination of quality care, and integration of ancillary services	Yes*	Yes	—^†^	Yes	Yes	Yes	Yes	—	Yes	Yes	Yes	Yes
Provider awareness and training	Guidance, training, and education for providers on treatment protocols and guidelines to standardize care, screen and refer for treatment, and increase familiarity with additional clinical or social service resources and relevant state-specific laws and policies (e.g., plans of safe care)	Yes	—	Yes	Yes	Yes	Yes	Yes	Yes	—	Yes	—	Yes
Data, monitoring, and evaluation	Monitoring the burden of substance use or misuse through analysis of surveillance data, evaluation of programs, and policy or quality improvement initiatives	Yes	—	—	—	—	—	Yes	—	—	Yes	—	Yes
Financing and coverage	Medical coverage, reimbursement, and billing strategies for treatment of opioid use disorder during and after pregnancy, for prevention efforts, and to sustain long-term care provision	—	Yes	—	—	—	—	—	—	Yes	—	Yes	—
Ethical, legal, and social considerations	Programs, policies, or policy amendments to address social stigma and legal considerations (e.g., mandatory reporting) that affect uptake, access to, and provision of clinical, substance use, and mental health services	—	—	Yes	—	—	—	—	Yes	—	—	—	—

**Abbreviations:** AK = Alaska; FL = Florida; IL = Illinois; KY = Kentucky; NV = Nevada; OH = Ohio; PA = Pennsylvania; RI = Rhode Island; TN = Tennessee; VT = Vermont; WA = Washington; WV = West Virginia.

* “Yes” indicates a state is working on strategies within the area of focus.

^†^ Dash indicates a state is not working on strategies within the area of focus.

**TABLE 2:** Existing barriers to and facilitators of addressing opioid use disorder among pregnant and postpartum women and infants prenatally exposed to substances — Opioid Use Disorder, Maternal Outcomes, and Neonatal Abstinence Syndrome Initiative Learning Community, state action plans,* 12 states,^†^ 2018

Focus area	Existing barriers and facilitators
**Access to and coordination of quality services**	
Existing barrier	• Limited access to comprehensive clinical services, longer term MAT, and mental and behavioral health therapy because of limited number of specialized providers, delay in connection to care, variable transportation resources, and patient cost of services and treatment
	• Limited access to services in rural areas because of reduced provider and social service availability, constrained health care infrastructure, and patient distance from care
	• Lack of comprehensive, coordinated, quality, continuous, and integrated care systems and social services for women with OUD and infants prenatally exposed to substances during care transition (e.g., from prenatal, obstetric, and delivery/neonatal intensive care unit to postpartum and pediatric care; from positive screen for OUD to treatment)
Existing facilitator	• Telemedicine to facilitate access to care in areas with low provider capacity
	• PQC infrastructure to facilitate provider coordination of services
	• Existing facility-based interventions or in-patient programs with resources on parenting and social skills for women with OUD and infants prenatally exposed to substances
	• Existing care and service referral processes for infants prenatally exposed to substances, including infants with NAS to ensure connection to appropriate care and services
	• Existing workgroups or task forces to focus on health and social services for infants prenatally exposed to substances, including infants with NAS
**Provider awareness and training**	
Existing barrier	• Lack of statewide provider awareness and experience with identifying and treating OUD, being a MAT prescriber, linking patients to other trained MAT providers, or broader issues affecting use or misuse of substances
	• Inconsistent access to training and education for providers to better care for women with a positive screen for mental health conditions or substance use or misuse
	• Unclear reporting requirements and inconsistent application of evidence-based standards of care, including variable use of SBIRT for mental health or substance use or misuse in clinics and facilities
Existing facilitator	• Statewide 24-hour telephone support lines to support provider knowledge of MAT prescribing guidelines
	• PQC infrastructure to provide training opportunities (e.g., care bundles or waiver trainings)
	• Use of the SBIRT practice for provider training on mental health conditions and substance use
	• Leverage of current grant-funded programs to facilitate new training curricula for providers treating substance use
**Data, monitoring, and evaluation**	
Existing barrier	• Inconsistent data collection and monitoring practices within a state, affecting measurement of services, assessment of burden, and reporting (e.g., OUD prevalence among pregnant and postpartum women, and plans of safe care for infants and caregivers)
	• Limited in-state capacity to analyze data on prescription drug monitoring and OUD leads to delayed data analysis
Existing facilitator	• Existing statewide data systems that identify women who test positive for substance use during pregnancy and infants prenatally exposed to substances
**Financing and coverage**	
Existing barrier	• Variable coverage of MAT treatment and counseling, ranging from full to partial or limited coverage for services (e.g., coverage gaps beyond 6 weeks postpartum)
	• Limited provider understanding of insurance coverage for substance use treatment and counseling services, including MAT, which affects utilization of resources
	• Reimbursement issues, including lack of billing codes, coding discrepancies, and challenges with telemedicine or telehealth program reimbursement, resulting in limited provision of services
	• Lack of sustainable funding for programs, including PQCs, home visiting programs, screening and behavioral interventions, or drug treatment programs, that support quality care and services
Existing facilitator	• Current billing and reimbursement structures that incorporate OUD recovery treatment, including inpatient substance use treatment and services
**Ethical, legal, and social considerations**	
Existing barrier	• Stigma associated with substance use, including discrimination and criminalization
	• Fear of separation experienced by pregnant and postpartum women, from infants prenatally exposed to substance, including infants with NAS
	• Ethical concerns of health care providers about screening, reporting, and treating OUD during pregnancy
	• Gaps in provision of and access to social services, such as housing, transportation, and access to child care, for pregnant and postpartum women who use or misuse substances
	• Broader issues, such as polysubstance use, intergenerational poverty, and systemic factors and environmental conditions that might contribute to the opioid crisis that affect health outcomes
Existing facilitator	• Statewide substance use campaigns currently include antistigma messaging, and promote care coordination including plans of safe care for infants and caregivers

**Abbreviations:** MAT = medication-assisted treatment; NAS = neonatal abstinence syndrome; OUD = opioid use disorder; PQC = perinatal quality collaborative; SBIRT = screening, brief intervention, and referral to treatment.

* State action plans include an action document, presentation materials, and in-person discussions at the Opioid Use Disorder, Maternal Outcomes, and Neonatal Abstinence Syndrome Initiative Learning Community kick-off meeting in 2018.

^†^ Alaska, Florida, Illinois, Kentucky, Nevada, Ohio, Pennsylvania, Rhode Island, Tennessee, Vermont, Washington, and West Virginia.

**TABLE 3:** Strategies to address opioid use disorder among pregnant and postpartum women and infants prenatally exposed to substances — Opioid Use Disorder, Maternal Outcomes, and Neonatal Abstinence Syndrome Initiative Learning Community, state action plans,* 12 states,^†^ 2018

Focus area	Strategies
Access to and coordination of quality services	• Add a focus on pregnant and postpartum women and infants to statewide opioid initiatives and obtain internal state stakeholder confirmation
	• Communicate, collaborate, and coordinate within the state to avoid duplication of effort among agencies and organizations on OUD and NAS
	• Develop a MAT provider network map for pregnant and postpartum women with OUD using various state sources to share with stakeholders and the public
	• Implement evidence-based strategies to engage women in OUD treatment by building community-based service capacity to improve trauma-informed and family-centered care
	• Develop protocols and implementation processes for plans of safe care that include provision of services for postpartum women as caregivers for infants prenatally exposed to substances
	• Implement a PQC to coordinate OUD treatment networks, provide standards of care, disseminate communication and training on addressing stigma during care, and catalog social/wraparound services for pregnant and postpartum women and infants prenatally exposed to substances (e.g., nutrition and mental health services, housing services, parenting support, or early intervention)
	• Incorporate specific services and early education initiatives for infants prenatally exposed to substances into existing state frameworks and policies focused on infants and children
	• Improve care coordination and transition care from hospital discharge to pediatric services for postpartum women with OUD and infants prenatally exposed to substances
Provider awareness and training	• Educate providers and the health care community on the importance of MAT and counseling services
	• Educate providers and the health care community on requirements for plans of safe care requirements
	• Implement provider training on clinical standards and treatment using the prescription waiver to increase the number of active, listed, and licensed MAT providers
	• Train facility-based, prenatal, and community health care providers and program staff members on the SBIRT practice for pregnant women and caregivers of infants prenatally exposed to substances
	• Implement a PQC to develop clinical protocols, prescribing protocols, and standardized services for the treatment and management of pregnant and postpartum women with OUD, and the treatment and management of infants prenatally exposed to substances, including infants with NAS
	• Develop perinatal care practice standards and protocols for universal screening of prenatal and postpartum OUD, and facility-based screening for infants prenatally exposed to substances
	• Develop protocols for rapid quality improvement on care coordination of pregnant and postpartum women with OUD and infants prenatally exposed to substances
	• Develop a framework and training for implementing plans of safe care in all jurisdictions and communities
Data, monitoring, and evaluation	• Develop protocols to measure and evaluate rapid quality improvement on care coordination of pregnant and postpartum women with OUD and infants prenatally exposed to substances (e.g., PQC)
	• Develop a standardized data system to aid in identifying pregnant and postpartum women who use or misuse substances and infants prenatally exposed to substances, and collect information to meet Child Abuse Prevention and Treatment Act of 2016 reporting requirements
	• Identify standard data elements, data collection practices, and case definitions for OUD and NAS surveillance in birth hospitals
	• Establish a data-sharing process to identify eligibility for, referral to, and enrollment in special programs or social services for infants with NAS using data from multiple information systems to monitor early identification and connections to systems of care
Financing and coverage	• Identify and expand coverage to increase access to inpatient or residential OUD treatment and comprehensive services for postpartum women with infants
	• Collaborate with stakeholders to implement a care bundle for postpartum women with OUD and infants prenatally exposed to substances, including infants with NAS
	• Develop and implement a plan to provide and reimburse integrated, wraparound services for infants prenatally exposed to substances, up to age 1 year
	• Work with insurers, including Medicaid, to change prior authorization prescribing requirements for MAT, ensure full insurance coverage up to 1 year postpartum, and remove special requirements for prescribing approved medications
	• Identify sources for funding (e.g., Medicaid and federal grants) to support training efforts statewide and implementation of standardized clinical care
Ethical, legal, and social considerations	• Develop nonstigmatizing messages for providers of substance use prevention and treatment and social and child welfare services on support of pregnant and postpartum women with OUD and infants prenatally exposed to substances, including those with NAS
	• Train providers on implicit bias and antidiscrimination of pregnant women with mental health conditions or who use and misuse substances
	• Coordinate with statewide antistigma campaigns to address stigma toward pregnant and postpartum women who use and misuse substances, and infants prenatally exposed to substances
	• Standardize family-focused policies and practices across state agencies and health care organizations for postpartum women with OUD and infants prenatally exposed to substances

**Abbreviations:** MAT = medication-assisted treatment; NAS = neonatal abstinence syndrome; OUD = opioid use disorder; PQC = perinatal quality collaborative; SBIRT = screening, brief intervention, and referral to treatment.

* State action plans include an action document, presentation materials, and in-person discussions at the Opioid Use Disorder, Maternal Outcomes, and Neonatal Abstinence Syndrome Initiative Learning Community kick-off meeting in 2018.

**^†^** Alaska, Florida, Illinois, Kentucky, Nevada, Ohio, Pennsylvania, Rhode Island, Tennessee, Vermont, Washington, and West Virginia.

**Provider awareness and training**. Nine of 12 state team action plans focused on improving health care provider awareness and training related to care for vulnerable populations^¶¶^ (Table 1). Identified barriers included lack of awareness and experience among providers in identifying women with OUD and prescribing medication-assisted treatment to pregnant and postpartum women (Table 2). Strategies identified included implementing clinical protocols and standardized services; educating health care providers about evidenced-based screening and treatment standards; and developing plans of safe care (i.e., best practices for infants affected by substance use or withdrawal symptoms to ensure their safety and well-being once released from the hospital, and referral to services for caregivers, including mothers, with substance use disorder) requirements*** (Table 3). Resources such as screening, brief intervention, and referral to treatment training and provider 24-hour hotlines might facilitate efforts (Table 2).

**Data, monitoring, and evaluation**. Four of 12 state team action plans included establishing or modifying quality assurance and monitoring systems for vulnerable populations (Table 1). Reported barriers included inconsistent data collection and monitoring practices and limitations in data processing capacity (Table 2). Strategies included plans to develop quality improvement protocols, data systems, and standard data elements that identify pregnant and postpartum women with OUD and infants with NAS to improve care and service coordination (Table 3). Leveraging existing statewide data systems might advance implementation of data-related activities (Table 2).

**Financing and coverage**. Three of 12 state teams developed plans to address financing and insurance coverage (Table 1). Reported barriers were variable coverage of OUD treatment for pregnant and postpartum women and care of infants with NAS, issues with service reimbursement, and limited funding for services (Table 2). Strategies included collaborating with insurers and other stakeholders to expand coverage of services, implementing care bundles (e.g., groups of health services), limiting prior authorization requirements, and providing full health insurance coverage up to 1 year postpartum (Table 3). Modifying current billing and reimbursement structures might facilitate coverage of appropriate care for OUD (Table 2).

**Ethical, legal, and social considerations.** Two of 12 state teams focused on ethical, legal, or social considerations (Table 1). State teams reported that pregnant and postpartum women with OUD and infants with a diagnosis of NAS might experience stigma, including discrimination and criminalization, and gaps in provision of social services (Table 2). States noted that providers had ethical concerns about screening, reporting, or treating OUD during pregnancy because some states require reporting to child welfare or protection agencies.^†††^ State teams highlighted broader issues, including polysubstance use and systemic factors contributing to the opioid crisis. Strategies included creating nonstigmatizing messages for health care and service providers, training providers on unconscious bias and antidiscrimination practices for pregnant women with mental health conditions or OUD, and incorporating family-focused policies and practices into agencies and organizations (Table 3). Existing statewide efforts on substance use can be leveraged to improve care coordination and address stigma (Table 2).

## Discussion

OMNI LC aims to build state capacity to support systems change in states. Most states focused on increasing access to and coordination of quality services and provider awareness and training, with fewer states focused on data, monitoring, and evaluation; financing and coverage; or ethical, legal, and social issues. Implementing strategies to provide quality services and trained providers might be the initial areas of focus for states building capacity to improve perinatal outcomes for families affected by the opioid crisis. Future work in OMNI LC might focus on the importance of surveillance and evaluation, coverage, and stigma experienced by women and infants (*4**,**5*).

As has been found in other learning communities, stakeholder partnerships were identified by OMNI LC states as important across all focus areas and a necessary component of capacity-building (*6*). Stakeholder partnerships can act as levers to address barriers and are a critical aspect of implementing systems change (*6**,**7*). For example, states planned to engage hospital leadership, professional organizations, and provider champions in establishing statewide perinatal health networks.

Perinatal quality collaboratives are highlighted as a strategy and facilitator in the focus areas of access to and coordination of quality services and of provider awareness and training. These collaboratives are state-based networks for implementing quality improvement activities using rapid data analysis to improve the health of mothers and infants.^§§§^ Many state perinatal quality collaboratives address OUD and implement the patient-safety obstetric care bundle for pregnant and postpartum women with OUD, developed by the Alliance for Innovation on Maternal Health program.^¶¶¶^ The bundle includes developing partnerships with health care facilities and organizations, training providers on clinical care practices and standards, identifying state and local reporting guidelines, connecting women to appropriate care, and implementing requirements for plans of safe care.****

Beyond immediate care for pregnant and postpartum women with OUD, broader social and contextual issues discussed by state teams included lack of resources for mental health treatment, lack of sustainable funding for social programs, polysubstance use, and systemic factors such as intergenerational poverty. States noted difficulty with addressing OUD independent of other substance use (e.g., tobacco, alcohol, or marijuana). Approximately 90% of pregnant women who use opioids for nonmedical reasons concurrently use other legal and illicit substances (*8*), and with the changing nature of drug use, drug overdose deaths involving opioids, cocaine, or other psychostimulants are increasing (*9*). Social determinants of health, described as contributors to the opioid crisis, include intergenerational or persistent poverty, unstable housing, substandard education, and bias by race or ethnicity that might introduce stigma and unequal access to treatment and care (*10*). States in OMNI LC might focus on polysubstance use and additional social, ethical, and legal considerations, including the social determinants of health, by supporting multidisciplinary collaboration among various agencies (e.g., departments of housing, education, and public health).

The findings in this report are subject to at least three limitations. First, qualitative information collected reflects the activities and experiences of members of the state teams participating in OMNI LC. Thus, it is not representative of a state’s entire opioid crisis response activities, which might be directed by state priorities and available funding and capacity. Second, abstracted information sources required interpretation because verbatim transcripts were unavailable; however, the qualitative analysis protocol required consensus-based decision-making to limit over-interpretation. Finally, the findings of this analysis from 12 states are not generalizable to all states; however, strategies, barriers, and facilitators might be informative for states seeking to address the opioid crisis for vulnerable populations.

OMNI LC highlights strategies in five focus areas to address the needs of pregnant and postpartum women with OUD and infants prenatally exposed to substances and demonstrates the use of participatory multidisciplinary teams to identify possible strategies for intervention. By building capacity through statewide collaboration and leveraging of stakeholder partnerships (*6*), states might establish long-term, sustainable systems change and optimize maternal and child health outcomes.

**Summary**

**What is already known about this topic?**

Opioid use disorder (OUD) during pregnancy contributes to adverse maternal and infant outcomes, including neonatal abstinence syndrome. In response to the opioid crisis, changes in state-level systems are critical for improving health outcomes.

**What is added by this report?**

Multidisciplinary state teams most commonly identified strategies focused on increasing access to and coordination of quality services or improving provider awareness and training to improve outcomes for pregnant and postpartum women with OUD and infants prenatally exposed to substances, including opioids.

**What are the implications for public health practice?**

As identified by multidisciplinary state teams, implementing strategies to improve health care quality and training providers are important to addressing the opioid crisis. Future work with states’ teams might focus on increasing surveillance and evaluation, sustaining coverage, and reducing stigma experienced by women and infants.

## Footnotes

* CDC opioid portal site. https://www.cdc.gov/opioids/strategy.html.

† Twelve states were identified for participation in the first year of OMNI LC: Alaska, Florida, Illinois, Kentucky, Nevada, Ohio, Pennsylvania, Rhode Island, Tennessee, Vermont, Washington, and West Virginia. States were invited to participate in OMNI LC based on a high prevalence or incidence of opioid-related behaviors and outcomes (e.g., NAS incidence, OUD prevalence, overdose death rates), available treatment for OUD (e.g., medication-assisted treatment for pregnant and postpartum women), a declared state of emergency, and state-initiated or -developed interventions to address the opioid crisis.

§ https://www.acog.org/Clinical-Guidance-and-Publications/Committee-Opinions/Committee-on-Obstetric-Practice/Opioid-Use-and-Opioid-Use-Disorder-in-Pregnancy?IsMobileSet.

¶ https://store.samhsa.gov/system/files/sma16-4978.pdf.

** The following leaders participated on state teams: state health official; Medicaid medical director; behavioral, mental health, or alcohol and drug abuse director; Title V director; and a provider or facility champion. Each state team was composed of a minimum of five members representing the leadership described above. States might have included additional state staff members to support leadership participating in the meeting.

†† Participants of the in-person meeting included representatives of the following organizations: American College of Obstetricians and Gynecologists, Association of Maternal and Child Health Programs, Centers for Medicare & Medicaid Services, Health Resources and Services Administration, National Association of State Alcohol and Drug Abuse Directors, Substance Abuse and Mental Health Services Administration, Administration for Children and Families, and University of Illinois at Chicago.

§§ A strategy is defined as a method or technique used to enhance the adoption, implementation, and sustainability of a program, practice, or policy. Strategies should identify/define discrete components operationally, including: who enacts the strategy (actor); actions, steps, or processes, using active verb statements (action); and the target of the strategy (action target). https://www.ncbi.nlm.nih.gov/pmc/articles/PMC3882890/. Barriers and facilitators are defined as factors obstructing or enabling improvements, presenting problems or providing incentives, by moderating or mediating public health practice, programs, or policies.

¶¶ Vulnerable populations are defined in this report as pregnant or postpartum women with OUD and infants prenatally exposed to substances, including infants with NAS.

*** Plans of safe care are defined in the Comprehensive Addiction and Recovery Act of 2016, amended version (July 22, 2016). https://www.congress.gov/bill/114th-congress/senate-bill/524/text.

††† https://www.guttmacher.org/state-policy/explore/substance-use-during-pregnancy.

§§§ https://www.cdc.gov/reproductivehealth/maternalinfanthealth/pqc.htm.

¶¶¶ https://www.acog.org/About-ACOG/ACOG-Departments/Patient-Safety-and-Quality-Improvement/What-is-AIM?IsMobileSet.

****
https://safehealthcareforeverywoman.org/patient-safety-bundles/obstetric-care-for-women-with-opioid-use-disorder/ .
